# Impact of leg lengthening on viscoelastic properties of the deep fascia

**DOI:** 10.1186/1471-2474-10-105

**Published:** 2009-08-21

**Authors:** Hai-Qiang Wang, Yi-Yong Wei, Zi-Xiang Wu, Zhuo-Jing Luo

**Affiliations:** 1 Institute of Orthopaedics, Xijing Hospital, Fourth Military Medical University, Xi'an, 710032, PR China

## Abstract

**Background:**

Despite the morphological alterations of the deep fascia subjected to leg lengthening have been investigated in cellular and extracellular aspects, the impact of leg lengthening on viscoelastic properties of the deep fascia remains largely unknown. This study aimed to address the changes of viscoelastic properties of the deep fascia during leg lengthening using uniaxial tensile test.

**Methods:**

Animal model of leg lengthening was established in New Zealand white rabbits. Distraction was initiated at a rate of 1 mm/day and 2 mm/day in two steps, and preceded until increases of 10% and 20% in the initial length of tibia had been achieved. The deep fascia specimens of 30 mm × 10 mm were clamped with the Instron 1122 tensile tester at room temperature with a constant tensile rate of 5 mm/min. After 5 load-download tensile tests had been performed, the specimens were elongated until rupture. The load-displacement curves were automatically generated.

**Results:**

The normal deep fascia showed typical viscoelastic rule of collagenous tissues. Each experimental group of the deep fascia after leg lengthening kept the properties. The curves of the deep fascia at a rate of 1 mm/day with 20% increase in tibia length were the closest to those of normal deep fascia. The ultimate tension strength and the strain at rupture on average of normal deep fascia were 2.69 N (8.97 mN/mm^2^) and 14.11%, respectively. The increases in ultimate tension strength and strain at rupture of the deep fascia after leg lengthening were statistically significant.

**Conclusion:**

The deep fascia subjected to leg lengthening exhibits viscoelastic properties as collagenous tissues without lengthening other than increased strain and strength. Notwithstanding different lengthening schemes result in varied viscoelastic properties changes, the most comparable viscoelastic properties to be demonstrated are under the scheme of a distraction rate of 1 mm/day and 20% increase in tibia length.

## Background

The fascia consists of the superficial fascia and the deep fascia. The deep fascia is a dense connective tissue that lies beneath the superficial fascia. The fascia, not least the deep fascia, is of pointedly importance to surgeons, Orthopaedic surgeons, physiotherapists and orthotists. As a matter of fact, the deep fascia is closely linked with a multitude Orthopaedic diseases, which include congenital clubfoot [1], Dupuytren's disease [2] and scoliosis [3,4] for Orthopaedic surgeons. Given the continuum of connective tissue throughout the body, the mechanical role of the fascia and the ability of fibroblasts to communicate with each other via gap junctions, the fascia is likely to serve as a body-wide mechanosensitive signaling system with an integrating function analogous to that of the nervous system [5,6]. However, relatively few authors addressed chiefly the deep fascia, particularly the biomechanics of the deep fascia [7-9]. This is an important omission, owing to the general significance of the deep fascia in the body and the importance of biomechanics of the deep fascia. Actually, biomechanics has contributed to virtually each modern advance of medical science and technology [10]. In Orthopaedics, biomechanics has become an everyday clinical tool. Basic research has included not only in surgery [11,12], prosthesis [13,14], but in cellular and molecular aspects and healing in relation to stress and strain [15,16].

The biomechanical hallmarks of the deep fascia comprise nonlinear and viscoelastic behaviors, which include creep, stress relaxation, stress-strain hysteresis. The impact of mechanical loading on the deep fascia varies with time [10]. As well, uniaxial tensile test until rupture, which is carried out by loading and disloading with a constant tensile rate, is a common method to evaluate the mechanical properties of tissues. Such stress-strain tests symbolize the viscoelastic properties of certain tissues [8].

The concept of distraction histogenesis was introduced by G.A. Ilizarov [17,18]. Gradual distraction on living tissues creates stresses that can stimulate and maintain regeneration and active growth of certain tissue structures, which was named as the Law of Tension-Stress [17,18].

Despite the increasing number of studies addressing the impact of leg lengthening on a multitude of tissues, the response of the deep fascia has not been well documented. In the previous studies, we demonstrated the morphological changes of the deep fascia and subsequently provided direct evidence of regeneration of the deep fascia under certain regimen during leg lengthening [19,20]. However, no studies regarding the biomechanics of the deep fascia during leg lengthening have yet been reported to date and our knowledge. Nevertheless, the biomechanical properties of a certain tissue as well as those during leg lengthening are of critical importance to clarify the underlying mechanism of the principle of Tension-Stress. From this point of view, the purpose of this study was to evaluate the viscoelastic properties of the deep fascia during leg lengthening.

## Methods

### Establishment of leg lengthening animal model

In adult New Zealand white rabbits (License number SCXK 2002–005, lab animal center of the Fourth Military Medical University), the deep fascia in the leg were lengthened by a unilateral external fixator applied with four pins to the medial surface of the tibia [21]. The committee on animal experimentation of Fourth Military Medical University approved all experiments, which met the NIH guidelines for the care and use of laboratory animals. A monofocal proximal diaphysis osteotomy between the second and the third pins was performed with little incisions, the periosteum and the skin closed. Seven days after operation [22,23], axial distraction was initiated at two different rates, i.e., 1 mm/day and 2 mm/day. Lengthening was performed twice daily and continued until 10% and 20% increases in the initial length of the tibia had been achieved. Twenty adult New Zealand white rabbits were randomly divided into 4 groups, each of which included 5 animals. The animals were grouped as reported previously [19,20]. In brief, four leg lengthening schemes, i.e., 1 mm/d with 10% and 20% increment in the tibia length, 2 mm/d with 10% and 20% increment in the tibia length, correspond to group A, B, C and D, respectively. In a sham group of 2 animals the external fixator system was applied and osteotomy was made without lengthening.

### Tests of mechanical properties of deep fascia

The deep fasciae of different groups were excised from the gastrocnemius fasciae of legs of animals at various time points. All the mechanical tests were performed within six hours after the specimens were excised. During the period, the specimens were stored at 39.2°F in order to avoid mechanical property deterioration. Each fascia was cut into test specimens in dimensions of 30 mm × 10 mm (the initial length in the leg), and subsequently stored in normal solution at 39.2°F. The long axis of specimens was oriented parallel to the limb axis [24].

Each specimen was clamped at both ends on the Instron 1122 tensile tester (Instron 1122, Canton, MA). The air pressure upon the clamp was 58.6 × 10^6^N/m^2^, the aim of which was to avoid local slippage between the samples and the clamp. All the tests were carried out at room temperature with a constant tensile rate of 5 mm/min. The initial length of each specimen was 20 mm.

To determine the ultimate non-damaging loads applicable on fasciae, preliminary series of load-download tests were performed on specimens of different groups of animals on the basis of previous study on analogous tissues [9]. Given the ultimate non-damaging loads obtained from the preliminary series for control and experimental groups were different, the load for pre-conditioning for the hysteretic tests was 200 g for control fasciae; whereas the load was 400 g for the experimental groups.

Thirty-six fasciae specimens of experimental groups were involved in the current study as following procedures [8,10]. The direction of the strength was along the long axis of specimens, which was parallel to the limb axis. After being loaded with the pre-conditioning load, it was returned to its initial length. Then the specimens were re-stretched under the same load until five load-download cycles had been completed. As a result, the hysteresis curves were automatically generated. At the end of the hysteresis tests, fatigue tests were carried out by the elongation of the specimens until rupture [9,10].

### Statistical analysis

SPSS 11 for Windows (SPSS Inc. Chicago, IL) was utilized to perform the statistical analyses. The data of the stain at rupture (SR) and ultimate tension strength (UTS) was evaluated using analysis of variance followed by Student's t-test. Differences with P values of less than 0.05 were considered significant.

## Results

**Figure 1:**
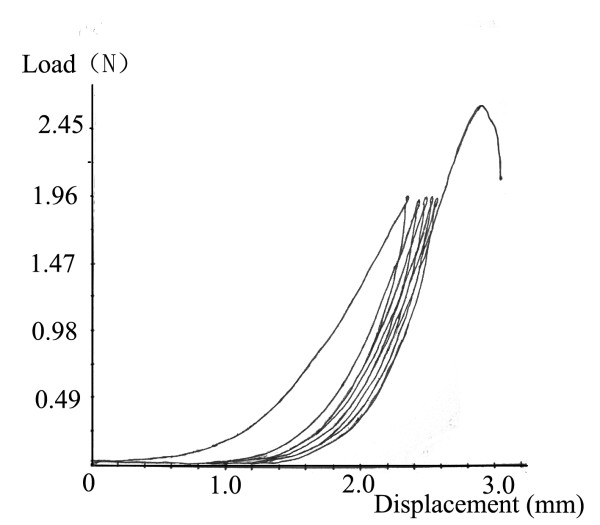
**The load-displacement curves of control fascia obtained from the hysteresis tests**.

**Figure 2:**
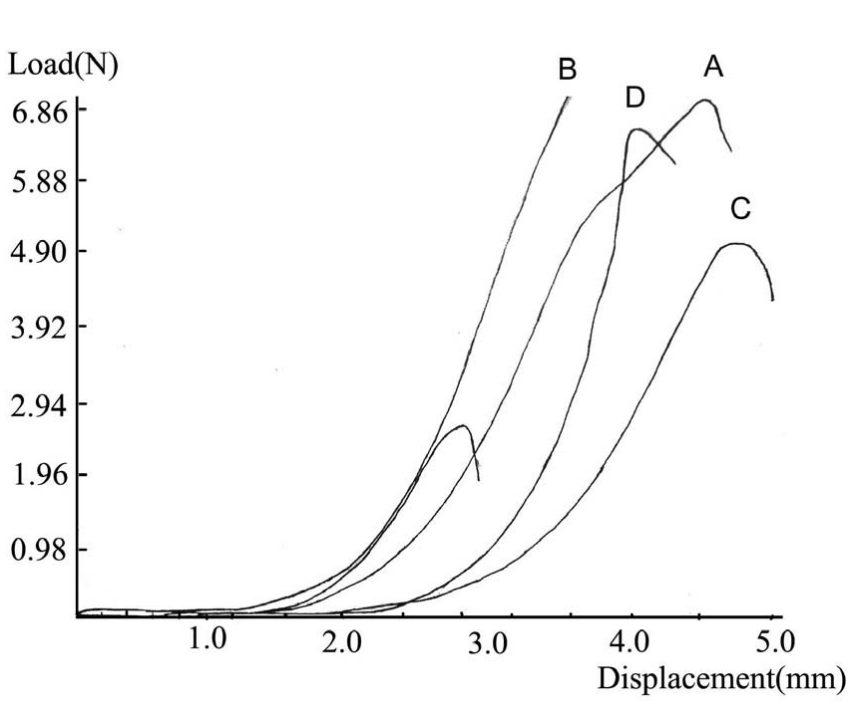
**The load-displacement curves of control and experimental groups produced by fatigue tests**.

Figure 1, the curve of control fascia, pointed to typical viscoelastic rule of collagenous tissues, i.e., the displacement increased rapidly at the initial stage of loading, during which the relationship between load and displacement was exponential. Then as the loading increased, the stiffness of material increased, during which the relationship was lineal. Figure 2 showed the load-displacement curves of control and experimental groups (i.e., A, B, C and D) produced by elongating the fasciae until rupture. Figure 1 displayed the load-displacement curve of control fascia of the uniaxial tensile tests. Figure 2 indicated that the curve of the fasciae subjected to the lengthening rate of 1 mm/day with 20% increase in tibia length was closest to that of normal fascia, even closer than at 10% increase in tibia length. As well, the curves of lengthening rate of 1 mm/d (A and B) were closer than those of 2 mm/d (C and D) to that of normal fascia.

**Figure 3:**
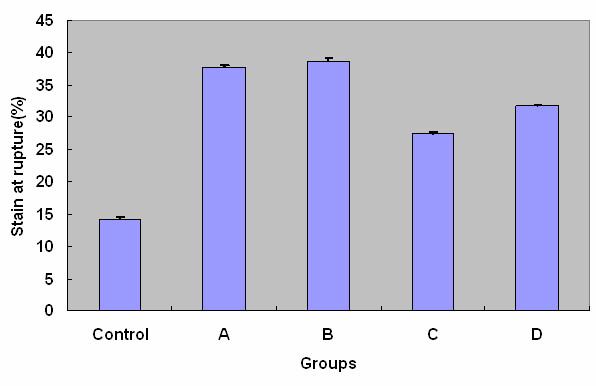
**The comparison of strain at rupture of each group**. The differences between control and each experimental group were statistically significant (*P *< 0.05).

**Figure 4:**
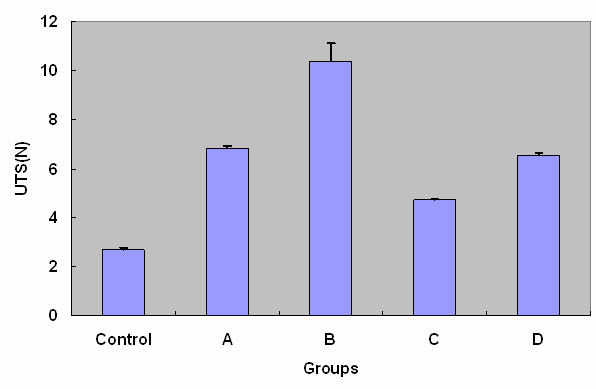
**The comparison of ultimate tension strength (UTS) of each group**. The differences between control and each experimental group were statistically significant (P < 0.05).

For the normal deep fascia without lengthening, the SR and UTS on average were 14.11% and 2.69 N (8.97 mN/mm^2^), respectively. After leg lengthening, the increases in UTS and SR of the deep fascia were statistically significant (*P *< 0.05). The comparison of SR and UTS of each group was demonstrated as Figure 3 and Figure 4, respectively. These figures indicated that SR and UTS increased statistically with the same order from maximum to minimum as group B, A, D, C and control.

## Discussion

Hitherto, this is the first study addressing the biomechanical properties of the deep fascia in response to leg lengthening. The biomechanical properties of the deep fascia were studied with hysteresis and fatigue tests and consequently load-displacement curves were generated and analyzed.

### Relationship between biomechanical properties and morphological changes of deep fascia

The results of both the hysteresis and fatigue tests indicate the constancy of viscoelastic properties of collagenous tissues as well as the alterations in load-displacement curves of the deep fascia after leg lengthening in comparison with that of the normal fascia. In addition, the curve of the deep fascia under the regimen of the distraction rate of 1 mm/day with 20% increase in tibia length was the closest to the normal one, even closer than the regimen of distraction rate of 1 mm/day with 10% increase in tibia length. As well, the curves of lengthening rate of 1 mm/d (A and B) were closer than those of 2 mm/d (C and D) to that of normal fascia. These biomechanical findings are undoubtedly linked to their corresponding structural or morphological changes [10]. A few studies regarding the morphological changes of deep fascia with various regimens of leg lengthening have been reported. Ilizarov [17] studied the morphological changes of various tissues of a canine model subjected to various distraction rates (0.5 mm, 1.0 mm, 2.0 mm) and distraction frequencies (one step per day, four steps per day, 60 steps per day). The fascia in its natural state had a wavy appearance when studied with the light microscope. Distractions at a rate of 1 mm once a day and in four steps, the collagen fibers had lost their wavy appearance and were oriented along the vector of distraction. While a distraction rate of 2 mm per day often resulted in undesirable dramatic changes within elongating fasciae. Recently, Stecco and colleagues [24] studied the organization of the deep fascia of the pectoral region and of the thigh in human from the view of surgical function. However, it is noteworthy that the deep fascia of various species and regions might have diverse morphological and functional hallmarks. Besides, we studied the morphological changes via light microscopy and electroscopy detection and the extracellular matrix alterations via histochemical methods of the deep fascia in the same distraction regimens as this biomechanical study [19,20]. Accordingly, normal deep fascia consisted of three layers under microscopy in the cross sections, i.e., two dense connective tissues outside and one loose connective tissue; whereas it comprised fibrocytes and collagenous fibrils in ultrastructure level [19]. As well, the appropriate regimen of distraction at the rate of 1 mm/d with 20% lengthening of tibia leads to the regenerative changes and the most comparable collagen composition in the deep fascia, more comparable than with 10% increase in tibia length. These morphological results concurred with and supported the findings of the present study, i.e., the deep fascia under the regimen of the rate of 1 mm/day in combination with 20% increment led to regeneration changes and consequently had a closest biochemical composition in comparison with normal deep fascia. Therefore they exhibited the closest biomechanical properties when compared to normal deep fascia.

Furthermore, our study showed that the SR and UTS between experimental groups were statistically different. The order of these values was B, A, D, C and control groups from maximum to minimum. The increment in strain and strength of the experimental groups might be attributed to the reorientation of collagen fibers along the vector of leg lengthening within the fascia [18]. In addition, the regeneration of fibroblasts and newly formed collagen fibers within the deep fascia might be a plausible explanation for the greater values under the lengthening rate of 1 mm/d (group A and B) than 2 mm/d (group C and D). However, we cannot find out a direct linkage between morphological changes with variations in strain and strength on the basis of the available evidence. Additional profound studies are needed to quantitatively connect the morphological and biomechanical changes and verify our findings.

Despite the progression on changes of the deep fascia during limb lengthening, the question of how mechanical stress turns into molecular or cellular signals remains open. It is believed that fibroblasts within the fascia are integral to mechanotransduction. They communicate with each other via gap junctions and respond to tissue stretch by shape changes mediated via the cytoskeleton [6,25]. As well, brief stretch decreases TGF-β1-mediated fibrillogenesis, which may be pertinent to the deployment of manual therapy techniques for reducing the risk of scarring/fibrosis after an injury [26]. Taking these studies into account, the alterations in the metabolic settings of fibroblasts, as the mainstay of mechanotransduction, within the deep fascia subjected to leg lengthening might contribute to the biomechanical changes. Therefore further studies are needed to clarify the accurate molecular mechanism of signal transduction during leg lengthening.

In our study, the SR of normal deep fascia was 14.11%, which coincides with the results of others. Yahia et al [8] studied the viscoelastic properties of human lumbodorsal fascia and noted that the SR varied from 12% to 15%. Other studies noted that the SR of the fascia lata of human was about 15% [9,27].

### Testing conditions of uniaxial tensile tests

The biomechanical properties of tissues are influenced by a variety of testing conditions, including the tensile rate [10], temperature [28,29], pH [30], ionic content [31] and sample geometry [32].

In our study, the testing conditions comprised a tensile rate of 5 mm/min and the room temperature of 55.76°F. The biomechanical properties of tendinous structures have traditionally been studied using isolated specimens via *in vitro *testing condition. Moreover, *in vivo *biomechanical detection can be extremely difficult and inconvenient even though it represents the function of tissues more accurately. Therefore the present study adapted the traditional *in vitro *testing condition.

Whereas the deep fascia is thin and smooth, it is essential to avoid slippage during uniaxial tensile tests. Various noncontact and *in vivo *tensile measurement methods have been reported [7,33,34]. Some authors studied the tensile properties of human soft tissues via *in vivo *and noninvasive protocols, i.e., ultrasound [33,34]. Zernicke and colleagues [7] established a noncontact optical method based upon following the motion of multiple landmarks located on the tissue surface to determine the discretized surface strains. However, these methods were mainly appropriate to nonhomogeneity of the tendon and fascia strains during high rate tests. In our study, the tensile rate was 5 mm/min and the clamp with air pressure was used to avoid slippage. As a consequence, the contact clamping method of the current study may have limitations associated with specimens' displacement and clamping and uncertainties as to whether *in vitro *material represents intact tissues function. However, the majority of tissue ruptures occurred near the middle-third part within the fascia. The phenomenon verifies the contact clamping method as a reliable alternative to study the biomechanics of soft tissue as the deep fascia. This might be due partly to the relatively low tensile rate we utilized.

Taken together, the biomechanical properties of the deep fascia are influenced by the stress during leg lengthening. Both lengthening rate and frequency contribute to the changes. Further study is needed to investigate the transduction mechanism of mechanical stress factors and the signaling pathway mediating the response to mechanical force in the deep fascia.

## Conclusion

The deep fascia subjected to leg lengthening exhibits viscoelastic properties as collagenous tissues without lengthening other than increased strain and strength. Despite different lengthening schemes result in varied viscoelastic properties changes, the most comparable viscoelastic properties to be demonstrated are under the scheme of a distraction rate of 1 mm/day and 20% increase in tibia length.

## Competing interests

The authors declare that they have no competing interests.

## Authors' contributions

HQW and ZJL conceived of the study, participated in the design of the study and performed the statistical analysis. All authors carried out the experiments. HQW drafted the manuscript with the help of YYW and ZXW. All authors have read and approved the final manuscript.

## Pre-publication history

The pre-publication history for this paper can be accessed here:


